# “Vitamin D supplementation and bone health in adults with diabetic nephropathy: the protocol for a randomized controlled trial”

**DOI:** 10.1186/1472-6823-14-66

**Published:** 2014-08-12

**Authors:** Diana R Mager, Stephanie T Jackson, Michelle R Hoffmann, Kailash Jindal, Peter A Senior

**Affiliations:** 1 Department of Agricultural, Food and Nutritional Science, University of Alberta, Edmonton, AB, Canada; 2 Department of Pediatrics, University of Alberta, Edmonton, AB, Canada; 3 Diabetic Nephropathy Prevention Clinic, Alberta Health Services, Edmonton, AB, Canada; 4 Northern Alberta Renal Program, Edmonton, AB, Canada; 5 Department of Nephrology, University of Alberta, Edmonton, AB, Canada; 6 Department of Endocrinology, University of Alberta, Edmonton, AB, Canada

**Keywords:** Vitamin D supplementation, Bone health, Diabetes, Kidney disease

## Abstract

**Background:**

Suboptimal vitamin D status is highly prevalent in Northern communities, particularly in those patients with chronic diseases such as diabetes and chronic renal disease. Emerging literature suggests that adherence to daily vitamin D supplementation may be an important factor influencing vitamin D status and overall bone health, but compliance with therapies for bone health is a major challenge. It is unknown what level of vitamin D supplementation will ameliorate or improve suboptimal vitamin D status in patients with diabetic nephropathy or contribute to improved bone health, particularly for those living in northern climates.

**Methods/Design:**

The study purpose was to examine two different strategies of vitamin D3 supplementation; daily dosing of 2000 IU per day verses monthly dosing of 40,000 IU per month on markers of vitamin D status, bone health and to examine whether adherence, quality of life and patient satisfaction with the supplementation strategy differs between the two vitamin D strategies in adults diagnosed with diabetic nephropathy.

**Discussion:**

The need for RCTs assessing higher doses of vitamin D_3_ supplementation at varying frequencies of administration and its impact on bone health in adults with diabetes and chronic kidney disease are needed.

**Trial registration:**

ClinicalTrials.gov NCT01476501.

## Background

Suboptimal vitamin D status (25(OH)D <75 nmol/L) is associated with the development and progression of both diabetes and chronic kidney disease (CKD) [1]. Within the general North American population 16-52% have suboptimal vitamin D status; the prevalence of vitamin D insufficiency increases to 86% in the diabetic population and those with concurrent kidney disease are 1.78-fold more likely to be vitamin D deficient [2-4]. Patients living in northern communities are at particular risk for vitamin D insufficiency due to limited sunlight exposure, further increasing their risk for low bone mineral density (BMD) and fragility fractures [3,5-10]. Recent evidence indicates that up to 40-90% of patients with stage 3–4 CKD have insufficient/deficient vitamin D status [2,5,11,12]. By the time CKD patients reach dialysis, approximately 75% have metabolic bone disease [13].

Unlike other nutrients, recommendations for vitamin D are not based on food sources as there are very few dietary options available (e.g. fish, liver and fortified dairy products), but rather are designed to compensate for a deficiency of sunlight [14]. This places the individual living in northern climates at particular risk for inadequate vitamin D status, especially in the winter months when sunlight exposure is unlikely to contribute to overall vitamin D status. Most evidence suggests that when vitamin D requirements are met, it is with a combination of dietary vitamin D and a supplement [15-22]. Patients with diabetic nephropathy are at increased risk for poor dietary intake of vitamin D due to restrictions on vitamin D rich foods/beverages (e.g. dairy based products) as these products also have a high carbohydrate, phosphorus and/or potassium content. It is unknown what level of vitamin D supplementation will ameliorate or improve suboptimal vitamin D status in patients with diabetic nephropathy or contribute to improved bone health, particularly for those living in northern climates [5,7,10]. A recent study in patients with stage 3–4 CKD demonstrated that daily oral supplementation of vitamin D_3_ (1,000 IU/d) for three months resulted in a mean increase in serum 25(OH)D of 25 nmol/L (40 ± 15 nmol/L pre- vs. 68 ± 25 nmol/L post-supplementation); a significantly lower level of 25(OH)D than is thought to optimize bone health (>100 nmol/L) [12,23-25].

Emerging literature suggests that adherence to daily vitamin D supplementation may be an important factor influencing vitamin D status, and compliance with therapies for bone health (like other asymptomatic conditions) is a major challenge [26-28]. Chronic diseases, such as poor bone health, as well as suboptimal vitamin D status, have been associated with reduced quality of life (QoL) [26,29-31]. Despite the potential for improved functional ability and independence, only 50-69% of individuals prescribed osteoporosis medications (e.g. bisphosphonates, vitamin D and calcium) comply to them regularly (e.g. consume 80% of the time), and only 25-35% are compliant for more than one year [29-31]. This suggests that current modes of vitamin D supplementation in adults with diabetic nephropathy, particularly low dose daily administration (<1,000 IU/d), may be ineffective at optimizing vitamin D status. Higher daily doses (>1,000 IU/d) or the use of high dose, less frequent modes of administration (monthly vs. daily), need to be explored to ensure improved compliance to dosing strategies and adequacy of overall vitamin D status, particularly in those populations at high risk for vitamin D insufficiency and suboptimal bone health (e.g. diabetic nephropathy).

## Purpose

The first study aim was to investigate the impact of daily vs. monthly vitamin D_3_ supplementation on vitamin D status and markers of bone health in adults with diabetic nephropathy. The second study aim was to compare participant adherence and satisfaction between two different vitamin D_3_ supplementation strategies (daily vs. monthly), and quality of life (QoL).

### Objectives

1. Examine the impact of two approaches to high dose, oral, vitamin D_3_ supplementation (2,000 IU/d vs. 40,000 IU/m) for 6 months on overall vitamin D status (25(OH)D and 1,25(OH)_2_D) and markers of bone turnover (bone-specific alkaline phosphatase, osteocalcin, N-telopeptide of type 1 collagen) in adult patients with diabetic nephropathy.

2. Examine daily vs. monthly vitamin D_3_ supplementation strategies in regards to adherence, patient satisfaction and quality of life in adult patients with diabetic nephropathy.

### Hypotheses

1. Vitamin D_3_ supplementation (2,000 IU/d vs. 40,000 IU/m) for 6 months will result in significantly improved overall vitamin D status and improved markers of bone health in adult patients with diabetic nephropathy. Serum 25(OH)D will increase by a minimum of 25–50 nmol/L post-supplementation. Markers of bone resorption will decrease and markers of bone formation will increase after 6 months of vitamin D_3_ supplementation when compared to baseline levels.

2. Monthly dosing of vitamin D_3_ (40,000 IU/m) over 6 months will result in improved patient adherence and satisfaction with vitamin D_3_ supplementation when compared to daily dosing of vitamin D_3_ (2,000 IU/d) resulting in improved overall vitamin D status, bone health parameters, and quality of life.

## Methods

### Study design

The study design was a randomized, controlled, open-label trial comparing the effectiveness of two vitamin D_3_ dosing strategies (monthly vs. daily) on vitamin D status and markers of bone health in adults with diabetes and nephropathy over a 6 month period. Patients were randomized to one of the two vitamin D_3_ supplementation strategies in blocks of 30 using a random number generator (http://www.randomizer.org) by one graduate student (SJ): once monthly (40,000 IU/m; n = 60) or once daily (2,000 IU/d; n = 60) for six months. All members of the research team (with the exception the graduate students (SJ/MH)) were blinded to study allocation. Both vitamin D supplements contain vitamin D_3_ (cholecalciferol) in gel capsule form; Jamieson Natural Sources® Vitamin D 1,000 IU Soft gel (NPN 80017530), and EURO-Pharm International Canada Inc.® EURO D 10,000 IU (DIN 02253178). The two dosing regimens were: 2 capsules of 1,000 IU vitamin D daily (total dose = 2,000 IU/d), or 4 capsules of 10,000 IU vitamin D at the end of each month (total dose = 40,000 IU/m). The daily dose was selected based on results from findings that showed that patients with diabetes with stage 3–4 CKD in Northern Alberta where supplementation with 1000 IU/D vitamin D3 resulted in serum increases of 25(OH) vitamin D less than 25 mmol/L [5,7]. A monthly dose of 40,000 IU/m vitamin D_3_ was chosen to achieve equivalent supplementation to daily dosing assuming an adherence rate of 69%, and with the goal of obtaining a serum 25(OH)D concentration of 100 nmol/L [27,32].

This study was approved by the Human Research Ethics Board at the University of Alberta (File Number: Pro00022639), has received a “No Objection Letter” from Health Canada (Control File Number 148625), and is a registered clinical trial (NCT01476501). The study was monitored by a Drug and Safety Monitoring Board (DSMB) with annual safety reports submitted as per Health Canada protocol. Reporting of study results will be according to the CONSORT (Consolidated Standards of Reporting Trials) Guidelines [33].

### Participants

Patients were recruited from Northern Alberta Renal Program (NARP) clinics at Alberta Health Services (AHS) in Edmonton, Alberta between November 2011 and December 2013. This is a multidisciplinary program (endocrinologists, nephrologists, registered nurses (RN), registered dietitians (RD), pharmacists and social workers) that provides care to over 1,500 patients with diabetic nephropathy in northern Alberta. Sixty adults with diabetes and nephropathy per vitamin D_3_ supplementation group were recruited into this study (n = 120 total). Potential participants were approached by a member of the clinical team (e.g. RD or RN) and asked if a research team member could discuss this study with them. If verbal consent was provided, then a research team member contacted the patient, explained the study to them and determined their eligibility for participation in this RCT; if eligible and agreed by the patient, informed consent was signed and the baseline study appointment was booked. Subsequent study visits (e.g. 3 month and 6 month follow-up appointments) were booked via telephone calls made approximately 2 months later to follow-up with the participants and address any questions or concerns they may have had about their supplement strategy. Patient eligibility for this RCT was determined upon information available in the medical chart at time of screening. Inclusion/exclusion criteria are as follows:

### Inclusion criteria

1) Adult (18–80 years) patients diagnosed with diabetes (Type 1 and Type 2) and stage 1–4 CKD (Glomerular Filtration Rate (GFR) 15–89 mL/min/1.73 m^2^) [34].

### Exclusion criteria

1) Patients with co-morbid conditions known to affect vitamin D metabolism including gastrointestinal, liver, rheumatoid or bone disorders (e.g. hyperthyroidism, untreated celiac disease, cancer, Paget’s disease, sarcoidosis, malabsorption, etc.). Individuals with severe, permanent vision impairment will be excluded as this will preclude them from reading supplement labels accurately and safely. Pregnant women will be excluded as Dual-energy X-ray Absorptiometry (DXA) scans are not recommended during pregnancy. Patients weighing >136 kg will be excluded as the DXA table cannot accommodate this weight.

2) Patients on drug therapy known to interfere with vitamin D (e.g. oral glucocorticoids, cholestyramine, colestipol, mineral oil, Orlistat, digoxin).

3) Patients on other forms of active D metabolites (e.g. calcitriol, vitamin D2).

4) Patients with stage 5 CKD (GFR <15 mL/min/1.73 m^2^), receiving dialysis or on a kidney transplant list.

5) Patients with pre-existing hypercalcemia (>2.75 mmol/L), hyperphosphatemia (>2.0 mmol/L), severe secondary hyperparathyroidism (PTH >66 pmol/L), and serum 25(OH)D >200 nmol/L.

6) Patients with serum 25(OH)D <37.5 nmol/L at time of screening to control for correction of vitamin D deficiency [5].

7) Patients undergoing strict heavy exercise for weight control and/or those who used sunscreen lotion on a regular basis.

### Sample size

This sample size (n = 120) was based on the ability to detect a mean difference of 25–50 nmol/L in serum 25(OH)D from baseline levels after 6 months of vitamin D_3_ supplementation in each group (α = 0.05 and β = 0.8) with an additional 15% to account for potential subject attrition [5,8]. Recent evidence has shown that a mean increase of 25 nmol/L in 25(OH)D with 1,000 IU/day supplemental vitamin D_3_ was insufficient to promote 25(OH)D in excess of 100 nmol/L – the level of 25(OH)D that has been associated with having a beneficial impact on markers of bone health [5,7,23]. Therefore, we chose this specific vitamin D_3_ dose to ensure serum 25(OH)D would increase by 25–50 nmol/L.

### Data collection

Assessment of vitamin D status, bone health and lifestyle factors (diet, physical activity, sunlight exposure, QoL) were performed during study visits at the Clinical Research Unit (CRU) at the University of Alberta at baseline, 3 and 6 months post study enrolment between January 2012 and June 2014. Participants were given a 3 month supply of their respective vitamin D_3_ supplementation strategy at baseline and again at the 3 month study visit. They were asked to return their vitamin D_3_ vials/pill containers to the study investigators at the 3 and 6 month study visits. At baseline, information related to patient demographics and anthropometrics was collected. This included age, gender, ethnicity, medications/supplement use, insulin regimen, height, weight (Health O-Meter Professional model 597KL, Pelstar LLC, Alsip, IL, USA) and BMI. Changes in these variables between appointments were documented (e.g. medications, height, weight). Bone mineral density (BMD) was measured at baseline using Dual-energy X-ray Absorptiometry (DXA); a validated tool to assess BMD (General Electric LUNAR Prodigy, version 10.5, Madison, WI, USA). Whole body scans as well as site specific scans of the lumbar spine (L1-L4) and left total hip (including femoral neck) were conducted. The precision error, expressed as a percentage coefficient of variation (%CV), for the Lunar DXA located in the CRU are as follows: whole body BMD 0.7%, lumbar spine 0.9% and total hip 1.2%.

Lifestyle factors were assessed at baseline, 3 and 6 months using validated tools [35-41]. These include: 1) 3-day food record to assess vitamin D and calcium intake and other dietary factors known to influence vitamin D and bone health (phosphorus, carbohydrates, protein, caffeine). Dietary intake was analyzed using the Food Processor Database (Food Processor SQL, v.10.8, ESHA Research, Salem, Oregon, USA); 2) Weight-bearing physical activity records; 3) Sunlight exposure questionnaires; 4) Health related QoL (SF-36) questionnaires (http://www.qualitymetric.com Liscence Number QM019185 OPTUMInsight^TDM^); and 5) Adherence and acceptance surveys [34-38,42-44]. Adherence to vitamin D_3_ supplementation was also assessed by pill counts of returned vitamin bottles at 3 and 6 months.

### Laboratory investigations

To avoid risk of hypoglycemic events due to variations in appointment availability (e.g. insulin regimen incompatibility with fasting and later appointment times), random serum/plasma samples for measurement of routine clinical blood work and study blood work were collected. However, most blood samples were collected between 10 am-2 pm; approximately 2 hours post meal consumption (of either breakfast or lunch) to minimize the potential influences of variable blood collection times on bone turnover marker expression [39].

Blood samples were collected onsite at each research visit by a trained phlebotomist using validated techniques and tubes (SST gel for serum, and lithium heparin PST gel and EDTA plasma). Once collected, blood samples were immediately held at 2-8°C until processing by the research team or provincial laboratory system. Patients routinely receive clinical blood work to assess their glycemic control, kidney function and overall health, including: estimated GFR (eGFR), fasting/random blood glucose (FBG/RBG), urea, creatinine, hemoglobin A1c (HbA1c), calcium, albumin, phosphorus, magnesium, 25(OH)D and PTH. These variables were collected at all 3 study visits (except PTH) along with 1,25(OH)_2_D status. Serum PTH, bone turnover markers and fibroblast growth factor-23 (FGF-23) were collected at baseline and 6 month follow-up study visits.

Routine clinical blood work, 1,25(OH)_2_D and PTH were measured by validated, specific and sensitive methodologies used by the provincial laboratory system. Estimated GFR was calculated using a validated online equation provided by K/DOQI (Modification of Diet in Renal Disease (MDRD) study group and the Chronic Kidney Disease Epidemiology Collaboration (CKD-EPI) group; http://www.kidney.org/professionals/kdoqi/gfr_calculator.cfm). Bone turnover markers were analyzed by the research team using standardized commercial ELISA kits. After blood collection, EDTA plasma and serum clot samples (e.g. SST gel) were kept for approximately 30–60 minutes at 2-8°C and then centrifuged at 2,500 RPM at 4°C for 10 minutes (CR4.22 centrifuge, Jouan, Winchester, VA, USA). Recovered serum and plasma were aliquoted into clean micro-tubes according to volumes required for each specific assay to be tested in duplicate. Samples were stored frozen at −80°C until ELISA testing, and prepared according to the manufacturer instructions for each commercial ELISA kit: serum intact osteocalcin (OC; MicroVue Osteocalcin EIA Kit, Quidel, San Diego, CA, USA), serum bone-specific alkaline phosphatase (BAP; MicroVue BAP EIA Kit, Quidel, San Diego, CA, USA), serum N-telopeptide of type 1 collagen (NTx; Osteomark NTx Serum, Wampole Laboratories, Princeton, NJ, USA), and plasma intact fibroblast growth factor-23 (FGF-23; Human Intact FGF-23 ELISA Kit, Immunotopics Inc, San Clemente, CA, USA). The intra-assay (a) and inter-assay (b) coefficient of variance (CV) for these commercial kits are as follows: OC a) 4.8-10.0%, b) 4.8-9.8%; BAP a) 3.9-5.8%, b) 5.0-7.6%; NTx a) 4.6-13.99%, b) 6.9-13.99%; and FGF-23 a) 2.6-4.4%, b) 6.1-6.5%.

### Outcome measurements

**Figure 1:**
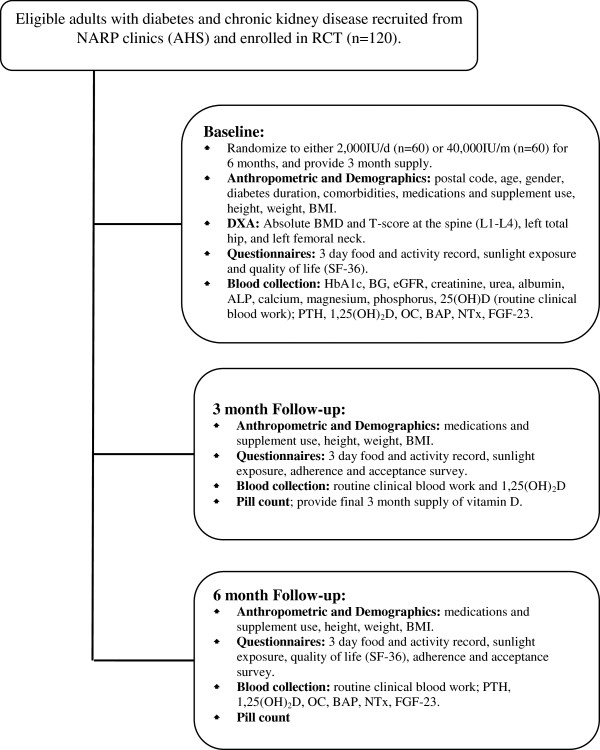
**Study design**^**1**^**. **^1^Abbreviations: Northern Alberta Renal Program (NARP); Alberta Health Services (AHS); randomized controlled trial (RCT); micrograms (mcg); Body mass index (BMI); Dual X-ray absorptiometry (DXA); Bone mineral density (BMD); Hemoglobin A1c (HbA1c); random blood glucose (RBG); estimated glomerular filtration rate (eGFR); 25-hydroxy vitamin D (25(OH)D); 1,25-dihydroxy vitamin D (1,25(OH)_2_D); alkaline phosphatase (ALP); osteocalcin (OC); bone-specific alkaline phosphatase (BAP); N-telopeptide of type 1 collagen (NTx); fibroblast growth factor-23 (FGF-23).

Outcome measurements are illustrated in Figure 1.

### Primary outcome variables

#### Serum 25(OH)D and 1,25(OH)_2_D

Serum 25(OH)D is considered the most reliable measure of vitamin D status as it accounts for cutaneous and dietary sources of vitamin D [40].

A mean increase in serum 25(OH)D of 25–50 nmol/L was chosen as a conservative target for 25(OH)D as 1,000 IU/d has been shown to increase serum 25(OH)D by 25 nmol/L in renal patients [5]. 1,25(OH)_2_D was measured to determine concentration of active vitamin D in participants and explore the relationship of active vitamin D with 25(OH)D levels and with bone health. Vitamin D levels were measured by validated, specific and sensitive methodologies used by the provincial laboratory system. The ratio of 1,25(OH)_2_D (product) to 25(OH)D (precursor) was calculated as a surrogate measure of 1α-hydroxylase activity (product to precursor ratio).

### Bone health

Bone health was assessed by measurement of BMD and plasma/serum levels of bone turnover markers. BMD was measured using DXA (non-invasive gold standard) to characterize bone health of participants at time of study entry. Bone turnover markers were measured at baseline and at 6 months; bone resorption: N-telopeptide type 1 collagen (NTx); and bone formation: bone-specific alkaline phosphatase (BAP) and osteocalcin (OC) [34]. Serum PTH and fibroblast growth factor-23 (FGF-23; measure of bone-kidney axis) were measured at baseline and 6 months to assess how serum PTH/FGF-23 concentrations change with our vitamin D supplementation strategies.

### Secondary outcome variables

#### Participant acceptance and adherence survey

Participants completed a validated survey developed to assess adherence to drug therapy in adults with chronic disease (e.g. hypertension) that has been adapted for our specific therapy [37]. This survey consists of open and close-ended questions. This was used to assess and compare adherence to the two different dosing strategies, and consider participants’ preferences and perceptions of their supplementation strategy. Adherence to the dosing regimens was also assessed by pill counts (at 3 and 6 month visits).

### Health-related QoL questionnaire (SF-36)

Participants completed the SF-36 QoL questionnaire (a common and validated tool used to asses QoL in chronic disease) at baseline and 6 months after study entry [38]. This questionnaire considers parameters of the participants’ functional capacity, social, and emotional well-being and was used to compare QoL pre-and-post intervention. (Liscence Number QM019185 OPTUMInsight ^TDM^).

### Dietary intake

3-day food records (2 weekdays, 1 weekend day), a validated tool for diet analysis, were completed [36,44]. Responses were verified by an RD on the research team (blinded to study allocation) and analyzed for micro- and macronutrient intake (e.g. calcium, vitamin D, phosphorus, carbohydrates, protein and caffeine) using Food Processor® (Canadian Nutrient File®).

### Weight-bearing physical activity

Analysis of the frequency/duration/effects of weight-bearing physical activity utilizing a record adapted from a validated physical activity questionnaire [34,42].

### Sunlight exposure questionnaire/seasonal affects

Participants completed a validated questionnaire for vitamin D synthesis potential according to sun exposure behaviours [43]. This questionnaire was completed to help account for seasonal variations and the impact this may have on vitamin D synthesis.

### Safety variables and analysis

Safety analysis included an evaluation of the literature and the potential for the following: 1) hypercalcemia [27,41,45] 2) signs and symptoms of acute toxicity (rare) including evidence “anorexia, nausea, vomiting, fatigue, confusion, headache, weakness, renal impairment, arrhythmias, hypertension, calcification of soft tissue and hyperphosphatemia” [41].

Adverse events with vitamin D_3_ supplementation at these dosing levels (40,000 IU/month or 2000 IU/D) have not been routinely reported in the literature [11,45-47]. Supplementation with active vitamin D analogues may be useful for individuals with severely diminished renal function and capacity for active hydroxylation, and is primarily used to treat severe hyperparathyroidism. However, providing already active vitamin D results in bypassing the homeostatic controls that are in place to prevent vitamin D toxicity (e.g. reducing 25-hydroxylation and increasing catabolism and excretion of 25(OH)D via bile), which in turn can increase risk for vitamin D toxicity [23,46]. Serum phosphate (>2.0 mmol/L), calcium-phosphorous product (CaP; >4.4 mmol^2^/L^2^) and magnesium (>1.0 mmol/L) are all monitored as part of routine clinical care for variation from healthy ranges [41].

A data safety monitoring board (DSMB) was included within the study design. All adverse events (AE) were documented. AEs were identified as related to the study protocol (e.g. anything directly related to our vitamin D supplementation intervention, blood collection or other examinations in the study protocol) or not related to the study protocol. Serious adverse events (SAEs) were defined as significant health/safety issues (e.g. acute renal failure, death). The responsible physician (PS), University HREB, and DSMB were notified of all AE/SAE. No SAEs due to study protocol occurred in this study.

#### Concomitant medication

Patients were asked to continue taking their normal medications as advised by their physician, e.g. insulin, oral hypoglycaemic agents, anti-hypertensives, statins, diuretics, phosphate binders, potassium lowering agents, anemia treatment, Replavite, and/or proton-pump inhibitors. Concomitant medications were reviewed at each visit to ensure excluded medications were not prescribed in the interim. Any patients medically requiring a concomitant medication that was contraindicated were released from the study. Participants were asked to discontinue all vitamin/mineral supplements containing calcium and/or vitamin D unless prescribed for therapeutic treatment (such as calcium carbonate-containing antacids for treatment of hyperphosphatemia). Those on active vitamin D treatment (with other vitamin D metabolites) were excluded from the study.

#### Rescue medication & risk management

Serum phosphate (>2.0 mmol/L), CaP product (>4.4 mmol^2^/L^2^) and magnesium (>1.0 mmol/L) were monitored throughout the study [48]. Vitamin D toxicity was addressed in the following ways: a) discontinuation of vitamin D_3_ supplement and b) notification to the Qualified Investigator (PS) and other members of the health care team in NARP/DNPC.

The following were standard responses to vitamin D toxicity in our population: 1) Serum and urine electrolytes, renal function, electrocardiogram, and fluid balance monitoring and maintenance [38]. 2) If necessary, additional measures may have been taken to enhance excretion/metabolism of the vitamin D including: IV administration of furosemide; corticosteroid, bisphosphonate or calcitonin therapy; hemo- or peritoneal dialysis [43]. If hypersensitivity to vitamin D_3_ occurred which included anaphylaxis, then appropriate treatment with epinephrine and ventilation would have been provided as needed.

Adverse events were reported immediately to the responsible physician (PS) or the on-call endocrinologist/nephrologist was notified as per standard clinical care. Participants were taken to the University of Alberta Hospital, AHS if requiring immediate medical treatment.

#### Premature withdrawal

Participants were able to voluntarily withdraw from the trial at any time without any negative consequences to their clinical care. Any and all reasons for participant drop out were documented. Any individual demonstrating clinical signs and symptoms of vitamin D toxicity and/or an SAE related to the study protocol resulted in: 1) vitamin D_3_ supplementation discontinued, 2) responsible physician (PS) notified so the patient received treatment for toxicity (as above), 3) notification of the DSMB and Health Canada, and 4) patient participation in the study was discontinued. New medical diagnosis arising precluding the ability to continue in the study was an additional reason for premature withdrawal. This was assessed by the qualified investigator (PS).

### Statistical analysis

Data analysis was performed on an intention-to treat basis as well as a per-protocol basis. Analyses were performed using Microsoft Excel 2010 and Statistical Analysis Software (SAS; version 9.3 SAS Institute, Cary, NC, USA). Statistical significance was determined at p < 0.05. Continuous variables were expressed as mean, median, ranges, and standard error (SE) or standard deviation (SD). The differences between dosing type (daily vs. monthly) over the intervention period were assessed by repeated measures analysis of variance, followed by a post-hoc pair-wise t-test with Bonferroni corrections to assess for within and between group comparisons. Potential risk factors for the development of vitamin D insufficiency (serum 25(OH)D <75 nmol/L) and poor bone health (T-scores < −2 SD) in adults with diabetic nephropathy include bone turnover, vitamin D intake, vitamin D status (serum levels of 25(OH)D and 1,25(OH)_2_D), age, gender, ethnicity, severity of kidney disease, diabetic management/control, exposure to sunlight, level of activity, and psychosocial and lifestyle factors.

Continuous and categorical variables (e.g. 25(OH)D, lab parameters, BMD T-scores, adherence scores) were quantitatively analyzed, and open-ended questions regarding participants’ perceived facilitators and barriers were categorized into key themes. Bivariate and univariate analysis were done to assess the potential effect of these variables on vitamin D status and bone health (bone turnover markers).Variables shown to be associated with a poor vitamin D status were assessed using multivariate logistic regression models to determine risk for development of vitamin D deficiency and poor bone health. Regression analysis was also conducted to assess correlation between biochemical parameters (PTH, 25(OH)D, 1,25(OH)_2_D, calcium, phosphorus, magnesium, albumin, eGFR, RBG) and bone turnover markers. Analysis of variance was performed to assess for significant differences in vitamin D status and bone turnover markers over the intervention period in both groups. Regression analysis was performed with serum 25(OH)D and plasma PTH as categorical variables; e.g. 25(OH)D sorted by greater/less than 75 nmol/L and plasma PTH sorted by greater/less than 7.15 pmol/L, the optimal concentrations for bone health [5,26]. Where necessary, vitamin D was adjusted for potential confounding variables (e.g. age, gender, ethnicity, disease severity) using an analysis of co-variance. When adherence to daily vitamin D dosing regimen was greater or less than 69%, the variation in compliance was also accounted for in an analysis of co-variance. For variables demonstrating skewed distributions a logarithmic transformation was used to normalize the data.

### Data management and validation

Prior to data entry, these individuals underwent ethics training and evaluation related to protecting participant information and health, including online University of Alberta and National Institute of Health ethics training and testing, criminal record checks, and ensuring up-to-date immunizations. Source data was coded and kept in a locked filing cabinet within the Clinical Research Unit, University of Alberta. Electronic files were encrypted and kept in a password protected computer according to University of Alberta (Faculty of Medicine) encryption policy [49].

The electronic data was audited in a timely manner to ensure any discrepancies were addressed and the potential for future discrepancies was reduced. A variety of standard operating procedures (SOP) were developed to support data analysis. Discrepant results were compared with source records, and amendments were made to the electronic records as necessary. All data entry was cross verified by one trained volunteer/graduate student, along with the primary graduate students involved in the project.

## Discussion

Vitamin D is a nutrient of concern for individuals with diabetes and nephropathy, particularly for those living in northern climates with limited sunlight exposure. Dietary restriction of vitamin D-rich food sources (e.g. dairy products) is a common issue due to concerns around phosphorus, potassium and carbohydrate content. Avoidance of sunlight is also common due to instructions for concomitant medications. Vitamin D supplementation is required in this population, yet the most effective dose and most efficient dosing strategy is unknown. Low adherence rates to routine vitamin D supplementation is also an inherent challenge and therefore developing alternative approaches such as alterations in dosing frequency (monthly verses daily) is also an important consideration [26]. Previous studies have been limited by small sample size, short intervention duration or retrospective design. This study prospectively investigated the impact of two different oral vitamin D_3_ supplementation dosing strategies (2,000 IU/d vs. 40,000 IU/m) for 6 months on markers of vitamin D status and bone health in a large group of adults with diabetes and nephropathy.

Measurement of markers of dynamic expression of bone turnover, enables evaluation of changes over time versus assessment of static changes in bone physiology that could be observed via DXA [50] is a conferred added strength. Markers of collagenous bone resorption (e.g. serum NTx), bone formation (e.g. serum BAP and OC), and the bone-kidney axis/mineral homeostasis (e.g. plasma FGF-23) will allow for exploration of dynamic bone remodeling during vitamin D supplementation. These markers have also been suggested as valuable tools for assessing adherence to bone health therapies [51].

Conferred additional strengths to the study design include the evaluation of socio-demographic, co-morbidities, concomitant medication use and quality of life factors; all factors known to influence adherence to vitamin supplementation. Ongoing analysis will enable a comprehensive evaluation of both lifestyle and physiological factors that influence response to vitamin D supplementation in this population. Dietary intake will be analyzed using validated methodologies for its contribution to total vitamin D intake and status, as well as intake of other nutrients that are known to impact bone health (e.g. calcium, magnesium, phosphorus). Finally, participants were enrolled throughout two calendar years, thus accounting for seasonal variations in vitamin D status. They completed sunlight exposure and weight-bearing physical activity questionnaires at each appointment so that potential impact of sunlight exposure during the different seasons as well as indoor and outdoor activity can be assessed for impact on vitamin D status and BMD.

Increasing evidence suggests that current recommendations for vitamin D intake are inadequate, particularly for populations at risk for suboptimal vitamin D status, such as those with diabetes, kidney disease or living in northern climates [3,5,7,27]. Moreover, the need for RCTs assessing higher doses of vitamin D_3_ supplementation at varying frequencies of administration and its impact on bone health in this population is supported by the research community [3,12,27,35,52,53]. The present study will help fill this gap in the literature, and elucidate an appropriate vitamin D_3_ supplementation dose for improved vitamin D status and bone health in adults with diabetes and nephropathy [12,27,35,52,53].

## Abbreviations

AE: Adverse event; ALP: Alkaline phosphatase; AHS: Alberta health services; BAP: Bone-specific Alkaline Phosphatase; BMD: Bone mineral density; BMI: Body mass index; CONSORT: Consolidated standards of reporting trials; CKD: Chronic kidney disease; CRU: Clinical research unit; d: Daily group; DNPC: Diabetic nephropathy prevention clinic; DSMB: Data safety monitoring board; DXA: Dual energy x-ray absorptiometry; ESRD: End-stage renal disease; FBG: Fasting blood glucose; FGF-23: Fibroblast growth factor – 23; GFR: Glomerular filtration rate; HbA1c: Hemoglobin A1c; HREB: Human research ethics board; K/DOQI: Kidney disease outcomes quality initiative; m: Monthly group; NARP: Northern Alberta renal program; NTx: N-telopeptide of type 1 collagen; OC: Osteocalcin; PTH: Parathyroid hormone; QoL: Quality of life; RBG: Random blood glucose; RCT: Randomized controlled trial; RD: Registered dietitian; RIC: Renal insufficiency clinic; RN: Registered nurse; SAE: Serious adverse event; SAS: Statistical analysis software; SF-36: 36-item short form questionnaire; 1,25(OH)_2_D: 1,25-dihydroxyvitamin D; calcitriol; 25(OH)D: 25-hydroxyvitamin D; calcidiol.

## Competing interests

None to report. Vitamin D suppliers (Jamieson Natural Sources®/EURO-Pharm International Canada Inc.®) had no involvement in study design/implementation, statistical analyses or manuscript preparation.

## Authors’ contributions

DM: Study design, grant, ethics and Health Canada submissions, supervised data collection (SJ, MH), data analysis, co-wrote manuscript, reviewed all version of the paper. SJ: Study design, grant, ethics and Health Canada Submissions, data collection, co-wrote manuscript. MH: Data collection, reviewed and contributed to manuscript. KJ: Contributed to study design, subject recruitment, manuscript revisions and submission. PS: Contributed to study design, reviewed grant, ethics and Health Canada submissions, assisted with subject recruitment, data collection and reviewed and contributed to manuscript submissions. All authors read and approved the final manuscript.

## Pre-publication history

The pre-publication history for this paper can be accessed here:

http://www.biomedcentral.com/1472-6823/14/66/prepub
